# A mature macrophage is a principal HIV-1 cellular reservoir in humanized mice after treatment with long acting antiretroviral therapy

**DOI:** 10.1186/s12977-017-0344-7

**Published:** 2017-03-09

**Authors:** Mariluz Araínga, Benson Edagwa, R. Lee Mosley, Larisa Y. Poluektova, Santhi Gorantla, Howard E. Gendelman

**Affiliations:** Department of Pharmacology and Experimental Neuroscience, 985880 Nebraska Medical Center, College of Medicine, University of Nebraska Medical Center, Omaha, NE 68198-5880 USA

**Keywords:** HIV-1, Monocyte–macrophage, Humanized mice, Antiretroviral therapy, Viral reservoirs, T effector cells

## Abstract

**Background:**

Despite improved clinical outcomes seen following antiretroviral therapy (ART), resting CD4+ T cells continue to harbor latent human immunodeficiency virus type one (HIV-1). However, such cells are not likely the solitary viral reservoir and as such defining where and how others harbor virus is imperative for eradication measures. To such ends, we used HIV-1_ADA_-infected NOD.Cg-*Prkdc* ^*scid*^ *Il2rg* ^*tm1Wjl*^/SzJ mice reconstituted with a human immune system to explore two long-acting ART regimens investigating their abilities to affect viral cell infection and latency. At 6 weeks of infection animals were divided into four groups. One received long-acting (LA) cabotegravir (CAB) and rilpivirine (RVP) (2ART), a second received LA CAB, lamivudine, abacavir and RVP (4ART), a third were left untreated and a fourth served as an uninfected control. After 4 weeks of LA ART treatment, blood, spleen and bone marrow (BM) cells were collected then phenotypically characterized. CD4+ T cell subsets, macrophages and hematopoietic progenitor cells were analyzed for HIV-1 nucleic acids by droplet digital PCR.

**Results:**

Plasma viral loads were reduced by two log_10_ or to undetectable levels in the 2 and 4ART regimens, respectively. Numbers and distributions of CD4+ memory and regulatory T cells, macrophages and hematopoietic progenitor cells were significantly altered by HIV-1 infection and by both ART regimens. ART reduced viral DNA and RNA in all cell and tissue compartments. While memory cells were the dominant T cell reservoir, integrated HIV-1 DNA was also detected in the BM and spleen macrophages in both regimen-treated mice.

**Conclusion:**

Despite vigorous ART regimens, HIV-1 DNA and RNA were easily detected in mature macrophages supporting their potential role as an infectious viral reservoir.

**Electronic supplementary material:**

The online version of this article (doi:10.1186/s12977-017-0344-7) contains supplementary material, which is available to authorized users.

## Background

Combination antiretroviral therapy (ART) reduces plasma human immunodeficiency virus (HIV) to undetectable levels in the infected human host [1, 2]. Nonetheless, ART-induced suppressed viremia rapidly re-occurs after treatment interruption [3–5]. ART failures to “cure” viral infection lead to the establishment of a stable latent viral reservoir [6–9]. In attempts to define this reservoir studies were initiated for both HIV and simian immunodeficiency virus (SIV) infections. Viral nucleic acids were detected in CD4^+^ T cells from blood, lymph node and gut associated lymphoid tissues (GALT) during ART [10–24]. Target cells for viral latency were within central memory (T_CM_) and transitional memory (T_TM_) CD4^+^ T cell pools [25–30]. Infected T_CM_ cells first emerged during cell reconstitution within CD4^+^ T lymphocyte compartments after ART intervention [31–35] and were maintained during plasma virus reductions and immune reconstitution [34, 35].

T_TM_ cells harbor significant amounts of proviral DNA and release progeny virus during the course of infection. These infected cells are most prominent when CD4^+^ T cell numbers are depleted during advanced disease [36–39]. While broad research in this area has been completed for more than a decade including linkages to cytokine dynamics and other parts of immune microenvironment little is known about the tempo of cell transformation and viral replication patterns. Defining such virus-host cell interactions is critical for selecting treatment interventions that interfere with the self-renewal and persistence of latently infected cells, which can ultimately lead to viral eradication.

To study disease patterns seen after viral infection and immune suppression, we constructed NOD.Cg-*Prkdc* ^*scid*^ *Il2rg* ^*tm1Wjl*^/SzJ (NSG) mice reconstituted with CD34+ human stem cells (HSC) [10–13, 40–43]. The HIV-1 infected humanized NSG mice can mimic critical features of human disease and are suitable to explore antiretroviral and adjunctive therapies [10–13, 40]. Noteworthy limitations include the life span of the human grafts and the human cell recovery rates.

In the present report, humanized NSG mice were infected with HIV-1_ADA_ then treated with two long-acting (LA) slow-release antiretroviral drug (ARV) regimens. LA ARVs were employed to optimize drug pharmacokinetic profiles [44–46]. One regimen contained LA cabotegravir (CAB) and rilpivirine (RVP) and prodrug nanoformulations of both lamivudine and abacavir (NM3TC and NMABC) [45, 46]. Another regimen used CAB and RVP. For simplicity in this report, the regimens are designated as 4ART and 2ART, respectively. The mice were infected for 6 weeks followed by ART administration for a month. At the study conclusion, animals were euthanized, immune cells isolated and viral nucleic acids examined by PCR amplification.

CD4+ T cells were recovered in blood, spleen and bone marrow (BM) of all ART-treated mice. However, HIV-1 infection affected numbers of memory and regulatory CD4+ T cells and monocyte–macrophages with limitations made on broad numerical cell comparisons. However, all cell types were readily restored by the ART regimens. Viral DNA and RNA levels were reduced by ART intervention. Interestingly, infected monocyte–macrophage populations persisted in spleen and BM tissues and were readily detected even considering rates of human cell reconstitutions. These findings, taken together, provide a model for future investigations that seek to target the ultimate elimination of the viral reservoir.

## Methods

### Animals and HIV-1 infection

New-born NSG mice were transplanted with human CD34+ stem cells obtained from human umbilical cord blood as previously described [13, 47]. After 15 weeks of reconstitution by human CD34+ HSC, mice were selected for further study based on their repopulation with human CD45+ cell numbers as monitored in peripheral blood. These animals were then infected with the macrophage tropic virus HIV-1_ADA_ by intraperitoneal injection at 10^4^ tissue culture infective dose 50 (TCID_50_)/mouse. Virus was prepared by infecting human monocyte-derived macrophages, then tittering the recovered virions as previously described [48]. After 6 weeks of infection, animals were randomly assigned to three groups; one group was treated with the 2ART regimen (HIV+2ART), one group was treated with the 4ART regimen (HIV+4ART), and one infected group remained untreated (HIV+). A fourth group of humanized NSG mice were uninfected and untreated, and served as negative controls (HIV−). After 4 weeks of treatment (10 weeks total following infection), animals were euthanized for immune cell phenotyping, flow cytometric sorting and virus detection in plasma and tissue. The 10 weeks total experimental time after infection facilitated the analysis and experimental studies and reduced the risk for great reduction of human cells in the transplanted animals.

### Quantification of plasma HIV-1 viral load (PVL)

Peripheral blood samples were collected into ethylenediaminetetraacetic acid (EDTA)-coated tubes from the facial vein and plasma was separated from blood cells by low speed centrifugation. The quantification of PVL of the virus-infected humanized NSG mice collected at 6 and 10 weeks post-infection was performed using the Taqman Analyzer according to the manufacturer’s instructions (Roche Diagnostics, Indianapolis, IN, USA).

### Antiretroviral drug (ARV) treatments

CAB, lamivudine (3TC) and abacavir (ABC) were obtained as generous gifts from GlaxoSmithKline (GSK) (Brentford, England, UK). CAB LA was prepared for use as a nanoformulation. 3TC and ABC were manufactured as prodrugs and encased in LA formulations by high-pressure homogenization [44–46]. RVP was purchased from Leap Labchem Co (HangZhou, China) and encapsulated into poloxamer 407 (Sigma Aldrich, St. Louis, MO, USA) nanosuspension [49]. Physicochemical parameters of each of the formulations including particle size, charge, polydispersity, shape and formulation stability were determined [44–46]. CAB LA and RPV LA were injected intramuscularly as a single dose at concentrations of 45 mg/Kg. NM3TC and NMABC were injected intramuscularly every week at concentrations of 40 mg/Kg. Drug treatment ended at 4 weeks when all animals were euthanized (Additional file 1: Figure S1). Drug concentrations in plasma, liver, lung, gut and or brain were quantified by ultra-performance liquid chromatography-tandem mass spectrometry during the course or end of treatment as previously described [10, 44–46]. Plasma values for CAB, RPV, 3TC and ABC were at or around the inhibitory concentration50 for each of the study drugs at the study end (data not shown).

### Peripheral blood and tissue collections

At study end, animals from the HIV-1, HIV-1 2ART or 4ART regimens and uninfected controls had their blood and tissue subjected to cell phenotype analyses. Blood samples were collected into EDTA-coated tubes by cardiocentesis at the time of euthanasia. Spleen and BM were collected in Roswell Park Memorial Institute (RPMI) media supplemented with fetal bovine sera (Thermo Fisher, Waltham, MA, USA). Recovered cells were processed to single cell suspensions for cell phenotype, sorting and viral quantification. Single cells from spleen and BM of HIV-1 infected NSG mice were separated into four aliquots for cell phenotyping, sorting, drug analysis and viral quantification. Tissues recovered included lung, gut, lymph nodes and brain were stored after recovery at −80 °C for subsequent viral quantification.

### Flow cytometry

For cell phenotyping, 100 μL of 10^5^ cell suspensions were acquired from whole blood, spleen and BM then incubated for 30 min with monoclonal antibodies to identify T cell subtypes, hematopoietic progenitor cells and monocyte–macrophages by flow cytometry [40]. Human monoclonal antibodies against CD45, CD3, CD4, CD8, CD45RA, CD45RO, CD95 and CCR7 identified frequencies of immunocyte populations. Memory CD4+ T cells were subtyped as stem cell memory (T_SCM_), central memory (T_CM_) and effector memory (T_EM_) with the expression of CD45RA+/CCR7+CD95+, CD45RO+/CCR7+ and CD45RO+/CCR7−, respectively, from the CD45+/CD3+/CD4+ gate. Moreover, CD4+ regulatory T cells (T_REG_) were classified as CD127+^low^CD25+, hematopoietic progenitor cells CD34 as lineage-CD34+ cells and monocyte–macrophages as CD3−/CD20−/CD8−/HLA-DR+/CD14++CD16+ cells. Flow cytometric acquisition and sample analysis was performed on a LSRII flow cytometer driven by the FACSDiva software package (BD Biosciences, CA, USA). All antibodies were obtained from BD Biosciences (San Jose, CA, USA).

Single cell suspensions were prepared from spleen and BM for cell sorting. Humanized NSG animal from all four groups were analysed from pooled cells and tissues. Human CD45+ cells obtained by magnetic beads were incubated with antibody panels for 30 min then used for flow cytometric sorting to isolate memory and regulatory cell populations, macrophages and CD34+ progenitor cells. The recovered cell populations included T_SCM_, T_CM_, T_EM_, T_REG_, total CD4+ T cells, monocyte–macrophages and hematopoietic progenitor cells. Each population were live-cell sorted in a designated BSL2 level biosafety cabinet using an Aria cell sorter (BD, Franklin Lakes, NJ, USA). During sorting, cell were collected in RPMI media, on ice, then pelleted by centrifugation and stored at −80 °C. Cell sorting was performed by the UNMC Flow Cytometry Core Facility. Analysis of all the acquired data was performed using FlowJo Version 10.08 software (TreeStar Inc., San Carlos, CA, USA).

### Nucleic acid extraction

Total viral nucleic acids extracted from tissue or from cells were acquired from the spleen, BM, lung, gut, lymph node, brain or from sorted cells using a Qiagen Kit (Qiagen, Hilden, Germany) according to the manufacturer’s instructions. Aliquots of eluted samples were used for PCR tests or frozen at −80 °C for later use. Total cell DNA and RNA obtained from the HIV-1 infected cell line ACH2 served as a positive control [40]. Human cells obtained from uninfected control animals were used as negative controls. Cell-associated HIV-1 RNA and DNA were quantified by droplet digital PCR (ddPCR).

### ddPCR for detection of HIV-1 nucleic acids

ddPCR test based on the water–oil emulsion droplet technology, was used for viral detection using specific primers and a TaqMan probe. For quantification of viral RNA, eluted cellular RNA was used as template for reverse transcription to synthesize first-strand cDNA using the iScript™ Reverse Transcription Supermix for RT-qPCR (Bio-Rad Laboratories, Hercules, CA, USA). The acquired cDNA was divided into two portions, the first for the detection of unspliced and the second for multiply-spliced viral RNA (usRNA and msRNA). The ddPCR assay for msRNA was performed with a primer pairs that amplified msRNA from the *tat* and *rev* genes [40, 50]. For usRNA tests, primers and fluorescent probes for amplification employed the HIV-1 *gag* gene region [40]. The ddPCR assay was run using the ddPCR™ Supermix for Probes reagents and in the QX200™ Droplet Digital™ PCR system (Bio-Rad Laboratories, Hercules, CA, USA). For the quantification of total and integrated HIV-1 DNA, the eluted cellular DNA was directly subjected to two rounds of PCR amplification. We modified a previously published protocol [50] for the amplification of total viral DNA (vDNA) targeting the HIV-1 *gag* gene. For the detection of integrated DNA (inDNA) we used an adapted *alu*-PCR assay [51] with modifications [40, 50]. The first round of the PCR was performed on a conventional PCR machine in 25 μL of PCR master mix, using the iTaq™ DNA Polymerase reagents (Bio-Rad Laboratories, Hercules, CA, USA) with specific primers [40]. The product of the first PCR was subsequently used as a template for the ddPCR amplification performed on the QX200™ Droplet Digital™ PCR system (Bio-Rad Laboratories, Hercules, CA, USA), using the ddPCR™ Supermix for Probes reagents and following the thermal cycling conditions for TaqMan detection chemistry as instructed by the manufacturer. Data acquisition and analysis were done using QX200 droplet reader and QuantaSoft™ software (Bio-Rad Laboratories, Hercules, CA, USA).

### Statistics

Univariate or bivariate homoscedastic data were evaluated by one- or two-way ANOVA, respectively, followed by appropriate post hoc tests. Nonparametric methods were utilized for non-normally distributed or heteroscedastic data. Data analyses were performed using Prism (v6, GraphPad Software, Inc., La Jolla, CA) and Statistica (v9, StatSoft, Inc., Tulsa, OK), and are presented within the text and figures as mean ± SEM.

## Results

### Viral replication in HIV-1 infected and ART-treated humanized mice

**Fig. 1:**
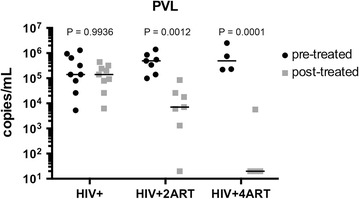
PVL quantification in the plasma of HIV-1 infected and ART treated humanized mice. Humanized NSG mice were infected with HIV-1_ADA_ and after 6 weeks of infection, VL was determined. Then, infected animals were distributed into three groups: untreated control, treated with 2ART or 4ART regimens. Plasma VL was measured after 4 weeks of treatment (10 weeks of HIV-1 infection). The figures represent viral copies/mL detected in plasma, using the COBAS Amplilink detection system. Two-way ANOVA for means (*bar*) of log_10_ copies/mL for 4–9 mice/group showed an effect of treatment by time (P = 0.00003). P values from pairwise comparison with Tukey’s HSD post hoc test are shown above each pre- and post-treatment pair groups

**Fig. 2:**
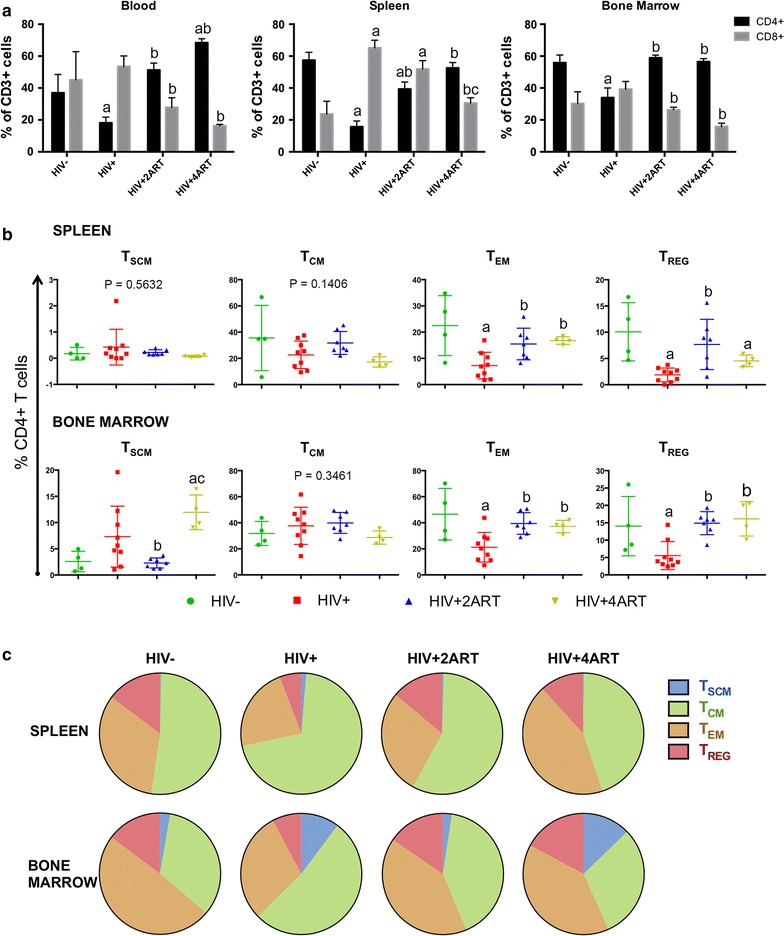
Lymphocytes phenotypes are illustrated during HIV-1 infection and ART treatment. Humanized NSG mice were infected with HIV-1_ADA_, and after 10 weeks of infection, cellular phenotyping were detected by flow cytometry. **a** First, percentages of CD4+ and CD8+ T cells were determined from total human CD45+ CD3+ gate from blood, spleen and BM of uninfected, infected and infected and treated animal with two or four ART regimens. Results shown are at 10 weeks post HIV-1 infection. **b** Then spleen and BM CD4+ cells were investigated to determine the phenotype for T_SCM_, T_CM_, T_EM_ and T_REG_ populations in all the groups as explained in **a**. Cell suspensions were labelled with anti-human monoclonal antibodies (mAb) targeting the following cell-surface markers: CD45, CD3, CD19, CD4, CD8, CD25, CD127, CD45RA, CD45RO, CD95, CCR7 (all from BD Biosciences). Data shows the percentage of the specific human CD4+ cells population. **c** Schematic description of the frequencies of T_SCM_, T_CM_ and T_EM_ and T_REG_ during HIV-1 infection and ART treatment in humanized mice. All acquisitions were performed on a LSRII flow cytometer (Beckman Coulter) and data were analysed by FlowJo software. **a**, **b** Comparisons of means (±SEM) for 4–9 mice per treatment group were determined by one-way ANOVA and pairwise significance by Fisher’s LSD post hoc test. P ≤ 0.05 compared with ^a^uninfected, ^b^infected, or ^c^infected and treated with 2ART

We examined two LA ARV regimens for their effects on infectious reservoirs within HIV-1 infected humanized mice. Four groups of humanized NSG mice were evaluated (Additional file 1: Figure S1). Virus was first assayed in plasma at 6 then at 10 weeks following viral infection (pre and post-treatment), respectively. VL in plasma from HIV-1 infected mice had on average 10^6^ copies/mL of virus at 6–10 weeks of infection. The viral copies/mL after the 2ART regimen was reduced to 10^4^ copies/mL and to undetectable levels in the 4ART group with one exception where one animal showed 10^3^ copies/mL (Fig. 1). The results showed that four ARVs could reduce VL at or below the limit of detection in plasma samples of infected humanized mice. *Second*, we tested cellular responses, altered as a consequence of HIV-1 infection and ART. The percentages of CD4+ T cells were evaluated 10 weeks after viral infection at study termination. Cell quantitation was performed 10 weeks after infection in blood, spleen and BM tissues from each of the mouse groups. As expected, we observed depletion of CD4+ T cells in blood, spleen and BM in the HIV-1 infected mice versus the uninfected animal group (Fig. 2a). Differences between the two animal groups were profound. CD4+ T cell percentages (mean ± SEM) were 18.1% ± 3.5 versus 37.0% ± 11.5 (in blood); 15.7% ± 3.7 versus 57.5% ± 4.9 (in spleen); and 33.9% ± 6.1 versus 55.9% ± 4.8 (in BM). The value differences were similar to those previously reported [40].

### CD4+ T cell subset frequencies

We recently showed changes in cellular subsets following HIV-1 infection [40]. Further investigation of these cells was initiated after ART and illustrated (Fig. 2a–c). CD4+ T cells recovered from spleen in the 2ART and 4ART regimens were restored to uninfected control levels (Fig. 2a). Frequencies of T_SCM_, T_CM_, T_EM_, and T_REG_ acquired from spleen and BM were evaluated following viral infection and ART. Interestingly, during ART, profound changes were seen in spleen and BM cells. In uninfected controls the frequencies of T_EM_ and T_REG_ were 22.4% ± 5.7 and 10.0% ± 2.7, respectively. During viral infection, these cell frequencies were reduced by 3- and 5-fold, 7.2% ± 1.7 and 1.8% ± 0.4, respectively, but recovered during ART. Frequencies of T_EM_ in 2ART and 4ART groups were 15.5% ± 2.2 and 16.8% ± 0.7, respectively, while T_REG_ frequencies were 7.7% ± 1.8 and 4.5% ± 0.5 cells, respectively. In BM, T_EM_ were reduced to half during infection, but recovered to uninfected levels with both ART regimens. For T_REG_, the frequencies were one-third of the uninfected controls, but reached uninfected control values with both antiretroviral regimens. No significant changes for T_SCM_ and T_CM_ frequencies were detected in spleen. However, in BM, T_SCM_ frequency increased after viral infection to 7.3% ± 1.9 versus 2.5% ± 0.9 in uninfected animals. With 2ART, T_SCM_ numbers were reduced in frequency at levels of uninfected controls, 2.3% ± 0.3, but reductions were not seen in the 4ART-treated animals, which showed 12.0% ± 1.6 cells (Fig. 2b). Such data supported the idea that the 4ART regimen stimulated cell subset production. A schematic chart showing CD4+ T cell frequencies in spleen and BM during HIV-1 and both ART regimens, as well as in uninfected controls, are shown in Fig. 2c.

### Monocyte–macrophages and CD34+ progenitor cell frequencies

**Fig. 3:**
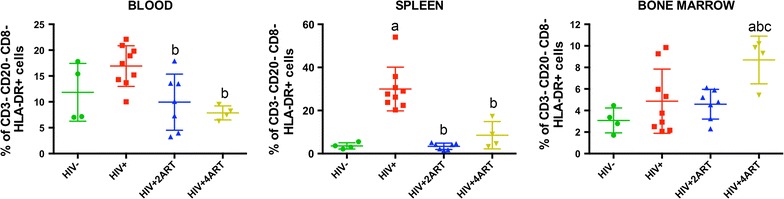
Monocyte–macrophage frequencies during HIV-1 and ART treatments. Humanized NSG mice were uninfected, infected, or infected and treated with 2ART or 4ART regimens. Cells from blood, spleen and BM were identified as CD3−/CD20−/CD8−/HLA-DR+/CD14+CD16+ by flow cytometry, as described in methods. Data were analyzed with FlowJo software. Mean ± SEM for 4–9 mice per treatment group were compared by one-way ANOVA, and pairwise comparisons were determined by Fisher’s LSD post hoc test. P ≤ 0.05 compared with ^a^uninfected, ^b^infected, or ^c^infected and treated with 2ART

**Fig. 4:**
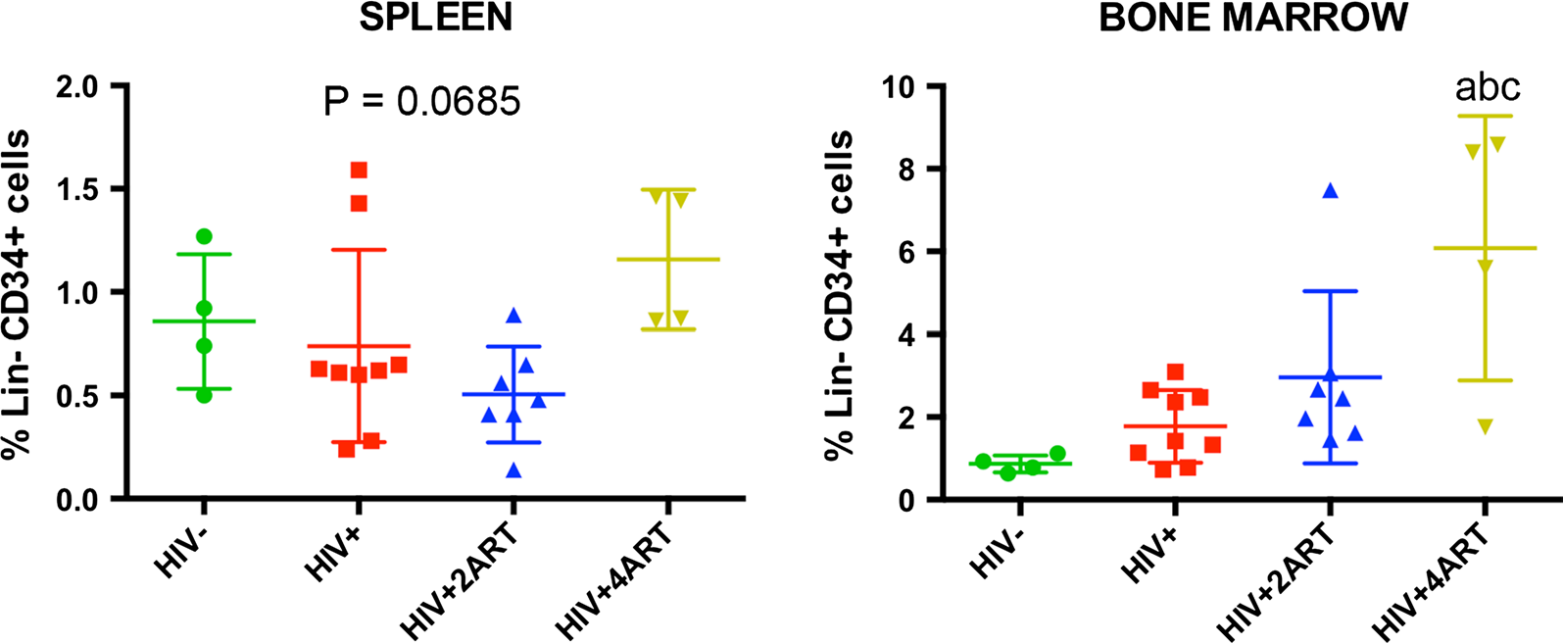
Identification of progenitor CD34+ cells. Multi-color flow cytometric analysis was performed on spleen and BM cells from humanized NSG mice that were uninfected, infected with HIV-1_ADA_ or infected and treated with 2ART or 4ART regimens. Cells from each tissue were incubated with labeled antibodies for the identification of CD34 and Lineage-1 cellular markers. Lin-CD34+ progenitor cells were determined by the expression of CD34 marker, excluding all Lineage-1 cells. Data were analyzed with FlowJo software. Mean ± SEM of 4–9 mice per treatment group were compared by one way ANOVA, and pairwise comparisons were determined by Fisher’s LSD post hoc test. P ≤ 0.05 compared with ^a^uninfected, ^b^infected, or ^c^infected and treated with 2ART

To determine how monocyte–macrophages and CD34+ progenitor cells phenotypes change during the course of infection and ART, we evaluated blood, spleen and BM cell frequencies at the study end. In monocyte–macrophages, HIV-1 infection resulted in cell frequencies of 17.0% ± 1.3 (in blood); 30.0% ± 3.3 (in spleen) and 4.9% ± 0.9 (in BM). In uninfected controls cell frequencies were 11.8% ± 2.7 (in blood); 3.5% ± 0.7 (in spleen) and 3.0% ± 0.5 (in BM). Differences seen during viral infection were altered by both ART regimens. Two and 4ART reduced monocyte–macrophage frequencies to 9.9% ± 2.0 and 7.8% ± 0.6 (in blood) and 3.4% ± 0.5 and 8.5% ± 3.1 (in spleen). The effect was not seen in BM from either two (4.6% ± 0.6) or 4ART (8.7% ± 1.1) during infection (Fig. 3). The frequencies for CD34+ progenitors cells from spleen were the same in all animal groups with 0.8% ± 0.1, 0.7% ± 0.1, 0.5% ± 0.1 and 1.1% ± 0.2 seen in infected animals with 2 and 4ART regimens and in uninfected controls. Interestingly, this was not observed for CD34+ progenitors cells from BM, where the frequencies were altered by HIV-1 infection compared to uninfected controls of 1.8% ± 0.3 versus 0.8% ± 0.1 and in 2ART and 4ART regimens (2.9% ± 0.7 versus 6.1% ± 1.6, Fig. 4). This result suggests that CD34+ progenitor cells turnover at a higher rate in BM during viral infection and ART.

### Viral nucleic acids identified in T cell subsets

**Fig. 5:**
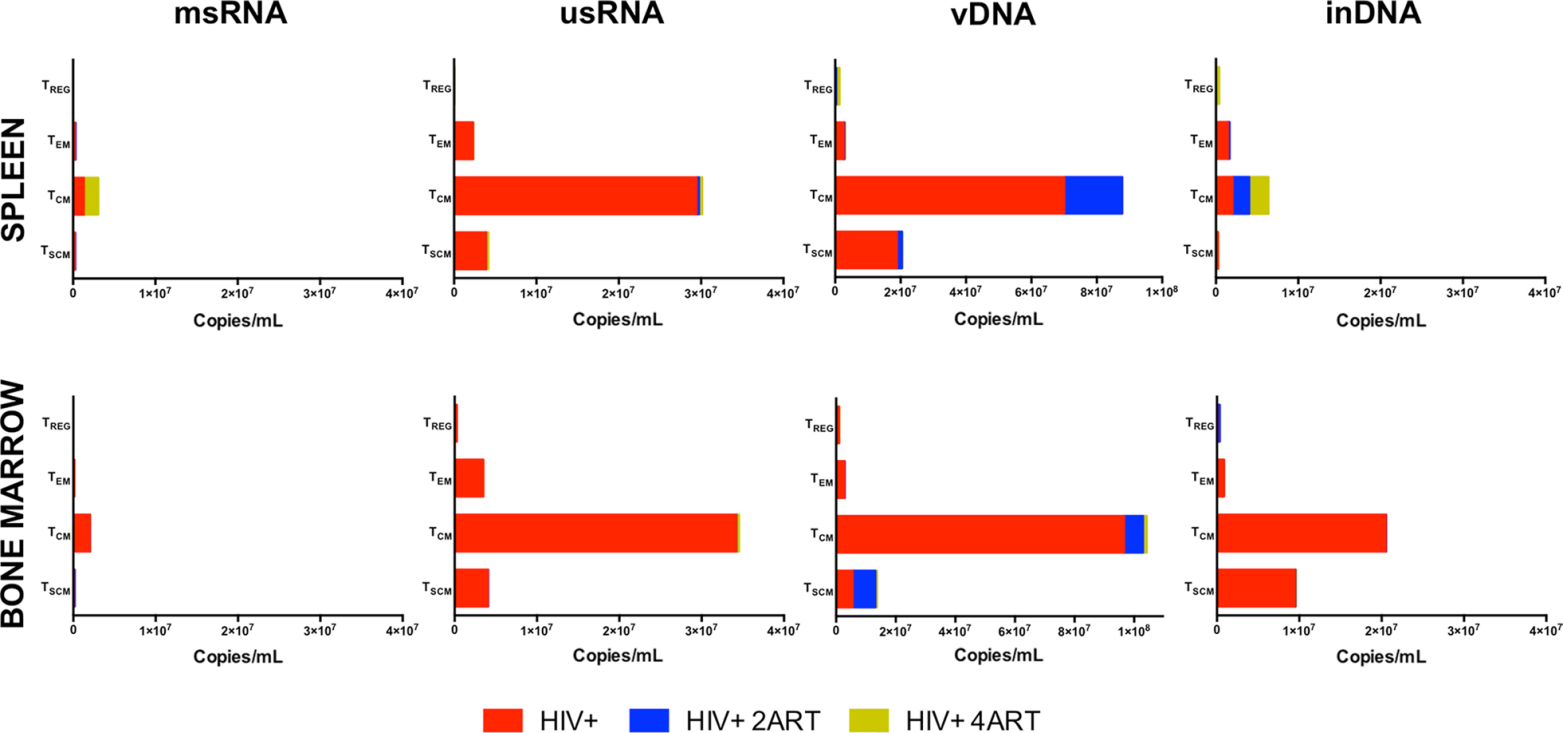
Defining the levels and species of HIV-1 infection in immune CD4+ subsets from spleen and BM, without and with 2ART or 4ART. Tissues were collected and cells in suspension were incubated with anti-human CD45 magnetic beads for isolating human CD45+ cells previous to FACS. Specific antibodies were applied and T_SCM_, T_CM_, T_EM_, and T_REG_ CD4+ cells were sorted. Then, sorted cells from 10 weeks HIV-1 infected humanized mice (4 weeks after ART) were used for detecting both viral RNA (msRNA and usRNA) and total viral DNA and inDNA as explained in methods, using the ddPCR system. The *numbers* indicate nucleic acid viral copies, RNA or DNA, per mL per each specific sorted subsets and frequencies for each group

To determine the cellular subsets that persist in HIV-1 infection and under antiretroviral therapy, we sorted by FACS and recovered several cell populations from pooled spleen and BM tissues of infected and treated humanized mice. Human CD4+ T cells were sorted for T_SCM_, T_CM_, T_EM_, and T_REG_ subsets. Following cell recovery, viral nucleic acids were assayed by ddPCR for detection of cell-associated HIV-1. HIV-1 msRNA and usRNA as well as total vDNA and inDNA levels were measured in all human T cell subsets. Viral RNA and DNA copies from sorted CD4+ T cells were reduced by both ART regimens (Fig. 5). Viral DNA and RNA were readily detected in T_SCM_, T_CM_ and T_EM_ cells following systemic infection. However, replicate cell types did not show significant msRNA, usRNA and vDNA after ART treatments. The ART effect was greater in the 4ART animal group; both in spleen or BM cells. However, inDNA was detected in human T_SCM_, T_CM_ and T_REG_ from spleens of mice treated with 4 ARVs (6 × 10^4^, 2 × 10^6^ and 3 × 10^5^, respectively), but not in BM. This suggested that infected CD4+ T cells from spleen remain resistant to ARV viral clearance. This was not seen in BM cells likely due to their high cell turnover that can serve to replenish virus-induced CD4+ T cell losses.

### HIV-1 persistence in monocyte–macrophages and CD34+ progenitor cells

**Fig. 6:**
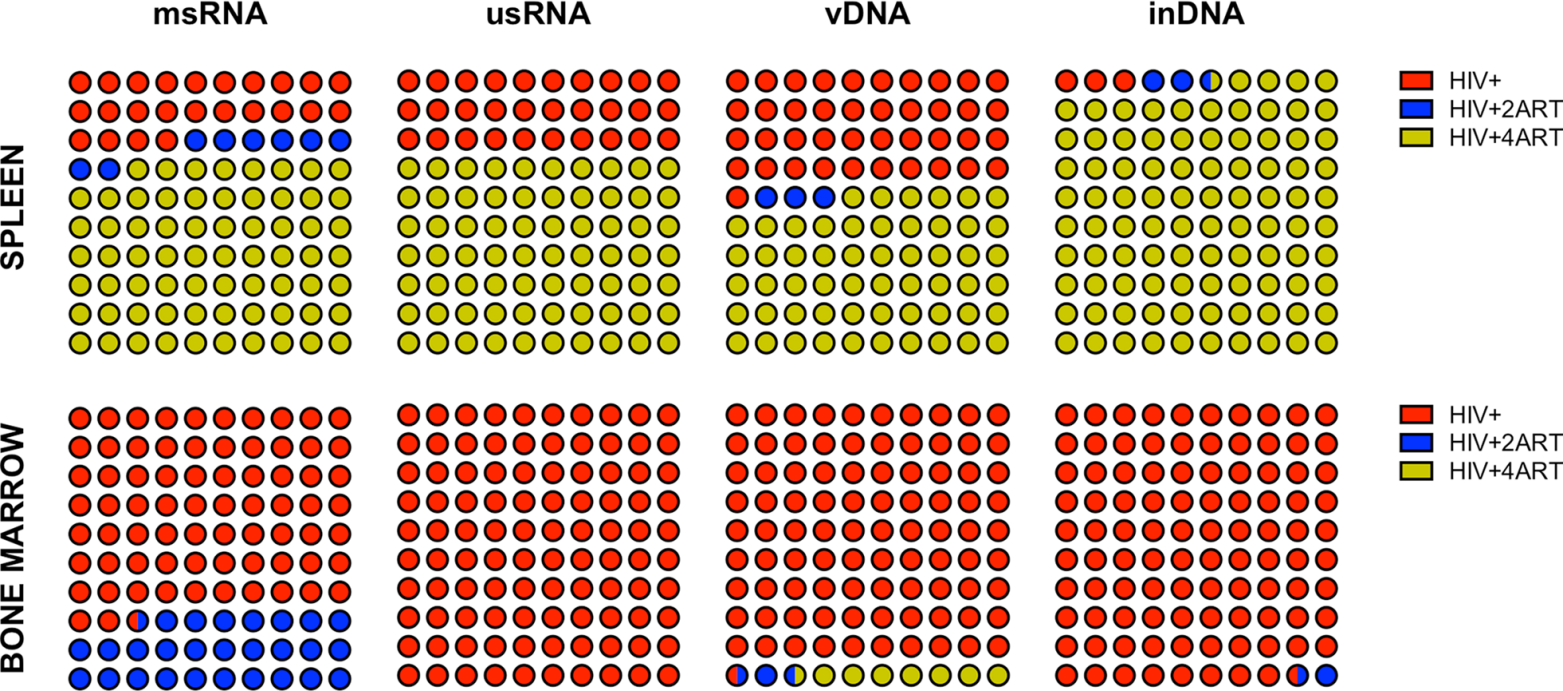
HIV-1 infection in monocyte–macrophages from spleen and BM and intervention of ART in the frequencies on infected cells. Sorted monocyte–macrophages CD14+CD16+ cells were processed for RNA and DNA isolation and examined by ddPCR system as described in methods. *Coloured dots* are representations for the frequency of viral RNA or DNA of different treatment groups from spleen and BM cells. *Dots in blue* indicate the HIV-1 infected control group, *dots in red* are the HIV-1 infected and 2ART drug-treated group and *dots in green* represent HIV-1 infected and 4ART drug-treated group

**Fig. 7:**
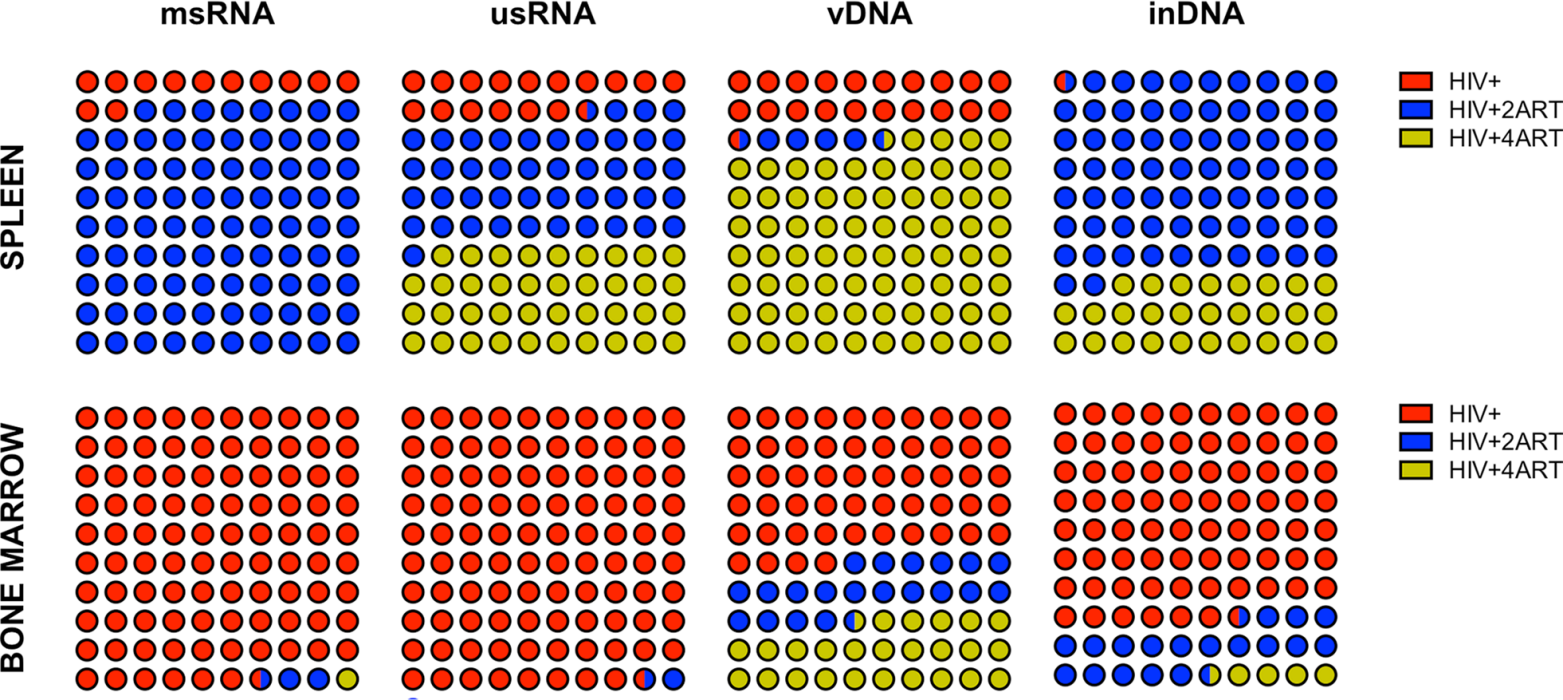
Frequency of infected progenitor CD34+ cells during HIV-1 with or without ART in humanized mice. At 10 weeks post HIV-1 infection, spleen and BM cells were sorted for Lin-CD34+ and were collected for RNA and DNA isolation for the detection of HIV-1 using the ddPCR system. *Coloured dots* are representations for the frequency of viral RNA or DNA of different treatment groups from spleen and BM cells. *Dots in blue* indicate HIV-1 infected control group, *dots in red* are for HIV-1 and 2ART and *dots in green* represent HIV-1 and 4ART regimens

Human monocyte–macrophages (Fig. 6) and CD34+ progenitor cells (Fig. 7) were immune sorted from pooled spleen and BM from humanized mice, then assayed for viral nucleic acids by ddPCR. Levels of viral usRNA and integrated DNA best reflect the pool of latently infected cells. Our results showed an unexpected pattern for human monocyte–macrophages from spleen, where viral clearance was not complete by either of ART regimens and was most prevalent in the group treated with 4 ARVs. Viral msRNA, usRNA, vDNA and inDNA copies/mL were even higher in the 4 ARV group reaching values of 5 × 10^3^, 3 × 10^5^, 8 × 10^5^ and 9 × 10^4^, respectively. However, all viral RNA and DNAs were reduced to nearly undetectable levels in human monocyte–macrophages from BM (10^1^, 10^1^, 10^3^ and 10^2^ copies/mL for viral msRNA, usRNA, vDNA and inDNA, respectively) (Fig. 6), which likely reflect more rapid cell turnover. CD34+ progenitor cells are known to be infected in HIV-1-infected humanized mice [40]. As shown after treatment with 2 or 4 ARVs, there was a significant virus reduction in BM cells (Fig. 7). Levels of integrated virus in BM cells were substantively reduced (<60 copies/mL). HIV-1 infected mice showed 3 × 10^2^ viral copies/mL in BM cells. However, this was not observed for CD34+ progenitor cells from spleen and perhaps the limited cell recoveries precluded complete analyses of viral clearance.

## Discussion

Research efforts directed at eliminating reservoirs of HIV-1 infection have focused on latently infected CD4+ T cell subsets [7, 52–55]. In addition to losses in CD4+ T cells along there is limitations in recruitment of virus-specific cytotoxic T lymphocytes. Both coincide with the emergence of latently infected T_CM_ [56–60]. Notably, a number of reports have shown that memory T cells are phenotypically altered during infection [31–35, 61, 62]. The altered CD4+ memory and regulatory cells occur during HIV-1 infection are recovered by ART. Our results from sorted cells of spleen are in accordance with previous reports demonstrating that T_CM_ cells are maintained during ART.

Monocyte–macrophages are an important reservoir for HIV infection. Such myeloid lineage cells are principal effectors of the innate immune system that engage multiple cell and tissue functions. This includes tissue homeostasis and repair to sensing and eliminating microbial pathogens and tumour cells (intracellular killing), secretion of bioactive factors and presentation of antigen [63]. Macrophage infection by HIV-1 was first acknowledged three decades ago [48, 64] and then after multiple studies have revealed infection of tissue macrophages [5, 65, 66] at all stages of disease, which persists under combination of ART [67]. Macrophages can promote HIV persistence and tissue damage, which has been demonstrated in lung, the central nervous system and spleen [59, 68–70]. In addition to producing infectious virus, spleen macrophages harbour latent proviral DNA and contribute as one important viral reservoir. Viral persistence in spleen macrophages is reflective of the cells’ long-half life. This affords the cells the potential to reactivate virus, continue to produce low level viral progeny, and be able to present infectious virus to T cells within the spleen and other lymphoid organs. Interestingly, we did not see this same cell behaviour for BM macrophages. Although the mechanism is not certain, the differences between these cells could reflect a faster turnover of macrophages in the BM, and as such make eradication much more difficult in these cells [71, 72]. This is based on the fact that monocyte turnover from bone marrow likely correlates with the development of end organ disease such as encephalitis and pneumonitis and occurs with signs of global activation in plasma typified by levels of CD163 and the actual size of the viral reservoir [73]. Certainly, determining how this occurs and the role different tissue macrophage populations play in viral persistence will require additional investigation.

While the present results provide new insights into how we view macrophage biology in relation to tissue location and HIV-1 infection we accept some notable limitations in both the model and approach. *First*, macrophage phenotype and function differs amongst its tissue of origin and hence its microenvironment and may differ in tissue or origin and species (mouse and man) [74]. Indeed, macrophages are a highly heterogeneous population with phenotypic characteristics defined by their environment. Whether spleen or BM phenotypic macrophage phenotypic variability might affect diverse ranges of tissue-dependent susceptibilities to HIV-1 infection and a spectrum of tissue responses that influence viral persistence, viral spread and pathogenic outcomes is now known [75]. Since the majority of studies performed on macrophages were based on blood-derived origins, our new findings likely represent specific tissue and disease specific cell biology made possible through this model for HIV-1 disease. *Second*, the human cell grafts are themselves time-limited. This could influence the establishment of complete viral latency. *Third*, as the numbers of human cells in tissue were dependent on graft cell infiltration and required cell pooling to perform the viral nucleic acid tests, the approaches themselves imposed limitations on statistical evaluations. *Fourth*, human cell reconstitution and the multiple time points evaluated posed limitations on the experimental design including viral quantification of the specific cell populations. Nonetheless, the investigations performed herein would not be possible in humans. These studies taken together provide, for the first time, insights into the role of subpopulations of human immune cells serve as viral reservoirs under combination long-acting ART in humanized mice.

The phenotypic changes of several immune cells affected during HIV-1 infection were previously found [40]. However, ART-affected phenotypic changes were seen in the present report and as such we were now able to determine populations of immune cells that enable persistent viral infection. Such cell populations are linked to on-going infection and viral spread in a spectrum of tissues. Indeed, differences seen between two and four ARVs also provide unique insights into the effectiveness of specific therapies. Even considering that ¼ of animals showed HIV-1 in plasma when receiving 4ART the treatment could serve to ultimately clear virus. This would occur in this animal if the time point was longer plus the dilution factor and estimated calculation required for plasma viral load analysis (copies/mL) was adjusted. Together, our findings show an important role of both memory CD4+ T cells and macrophage infection, specifically in spleen or BM. We posit that these long-live memory CD4+ T cells contribute to viral persistence. Macrophages capture and uptake infected CD4+ T cells and become infected and remains as reservoirs even under suppressive ART, as shown in our results by demonstrating the presence of integrated viral DNA in spleen macrophages, which can be a consequence of the ingestion of latently infected memory CD4+ T cells which also persist with any of both ART regimens. These particular events seems to be critical in spleen tissue, contrary to BM niche where there is a significant reduction of HIV-1-infected memory and regulatory CD4+ T cells, macrophages and progenitor hematopoietic cells. Cellular turnovers may dictate the capacity for viral infection and persistence, and differences in turnovers in peripheral blood, spleen and BM cells can definitely play a major role during HIV-1 infection during antiretroviral treatment.

To note, both ART regimens, used in this study, did not accomplished the capacity to clear infected central memory CD4+ T cells neither macrophages from pooled spleen tissue in our single experiment; perhaps differences could be noted by analysing single individuals rather than as a total group but we face the limitation of human cells recovery in this animal model. These reservoirs of infected cells will have the capacity to establish the infection and to carry integrated viruses that may contribute to disease progression. We posit that in differentiated tissue macrophages mature virions actively assemble and accumulate in invaginations of the plasma membrane becoming a virus-containing compartment that include late endosomes [76, 77]. Such virus-containing compartments cannot be reached by our ARVs thus begging the question of the native drug failures. Our group is currently focusing on the development of such long-acting ARVs and to target the same cellular compartments as virus [78] therefore facilitating improved treatments for viral clearance.

Results uncovered in this study that were not initially anticipated. One rested in the similar percentage of cell subsets seen between uninfected “control” mice and 2ART-treated mice, but not in 4ART-treated mice. Control and 2ART-treated infected mice had similar percentages of splenic and bone marrow memory and regulatory CD4+ T cells, but not early memory progenitors of CD4+ T and bone marrow Tscm cells. This suggested that the 4ART combination regimen induced cellular proliferation or high turnover of specific bone marrow populations. In support of this idea, spleen and bone marrow showed divergent behaviors. This paralleled our previously published data in humanized mice and was also found to be true for infected people when lymphocyte turn-over rates were evaluated after initiation of antiretroviral therapy [40]. *Second*, in a study using deuterated glucose to label DNA of proliferating cells, T cell dynamics in normal subjects and HIV-1-infected patients that were naive to antiretroviral drugs also showed divergent results amongst patients. Here rates of lymphocyte proliferation influence CD4^+^ T cell death [79]. Notably, CD8^+^ T cells proliferation rates are higher in infected people without mean death rates being influenced. *Third*, a parallel study that examined immune consequences following antiretroviral therapy demonstrated lymphocyte proliferation and death rates in CD4^+^ and CD8^+^ cell populations were linked to time, antiretroviral drug regimen, cell activation, end organ drug levels, gender, tissue type, and level of viral infection [80, 81]. *Fourth*, the largest decline was associated with effector memory and regulatory CD4+ T cells. Such changes were observed in all tested compartments. *Fifth*, the presence of stem cells in bone marrow and their relative expansion during infection and treatment could be affected by the types of ART and by differential responses seen by naïve cells. Thus, there are a number of potential explanations for why the 2ART and 4ART regimens behaved differently in these studies. Definitive tests are planned on human cells recovered from populations of murine bone marrow to test mechanisms of drug action, and as such, cell populations in divergent tissues are currently being re-evaluated. The second centered on the different impacts seen by ART in blood and spleen versus bone marrow. We posit that this could be explained by myelopoiesis which results in the constant generation of human monocytes. This process occurs only in bone morrow, but not in peripheral blood, and only at very limited levels in spleen. In this way expansion of macrophages that occurs during HIV-1 infection could be dependent on cell turnover rates. To this end, we now report changes in macrophage frequency of infection with and without ART. These findings paralleled a prior report that showed reductions in CD4^+^ T cell generation in bone marrow CD34^+^ progenitors during simian immunodeficiency virus infected macaques that resulted from changes in the clonogenic potential of marrow progenitors, which were of both myeloid and lymphoid lineage [82]. Hematopoietic failure was reported early in infection and in the absence of CD34^+^ infection and not linked to plasma viral load. Failure of hematopoiesis impairs T cell production in the bone marrow compartment, and as such, likely reflects tissue divergence. In the current study, our infected humanized mice showed higher frequencies of CD34+ cells during HIV-1 infection compared to uninfected control animals. Cellular frequencies were found affected by 2ART or 4ART treatment regimens. However, bone marrow cells did not respond the same as spleen cells as higher cell frequencies were observed during infection that did not return to a level observed in controls (uninfected, untreated animals). This suggested that once bone marrow is stimulated by infection, it results in higher cell frequencies. As virus populations in plasma and tissues differ significantly between treated and untreated infected animals suggests the exchange of virus or infected cells between tissue compartments. Thus, the differences are more likely reflective of cell type rather than differences in virus as viral replication during highly suppressive ART is limited [83]. Taken together, the ability of virus to stimulate cellular proliferation and perhaps cell turnover from bone marrow likely impacted different ART effects in blood, spleen, and bone marrow.

## Conclusion

We identified cellular reservoirs sites that persist after HIV-1 infection despite antiretroviral drug therapy (ART) in humanized mice. This study defines the HIV-1 cellular network in humanized mice and a potential cell source for viral persistence. The work serves as a model for future investigations to design antiretroviral therapies that target and ultimately clear cellular viral reservoirs.

## Additional file

**Additional file 1: Figure S1** Antiretroviral treatment scheme used in HIV-1 infected humanized mice.

## Abbreviations

HIV-1: human immunodeficiency virus type I
FACS: fluorescence-activated cell sorting
