# Supplementary figures and images for: A mature macrophage is a principal HIV-1 cellular reservoir in humanized mice after treatment with long acting antiretroviral therapy
Source: Retrovirology. 2017 Mar 9;14:17. doi: 10.1186/s12977-017-0344-7 (PMC5345240; doi:10.1186/s12977-017-0344-7)

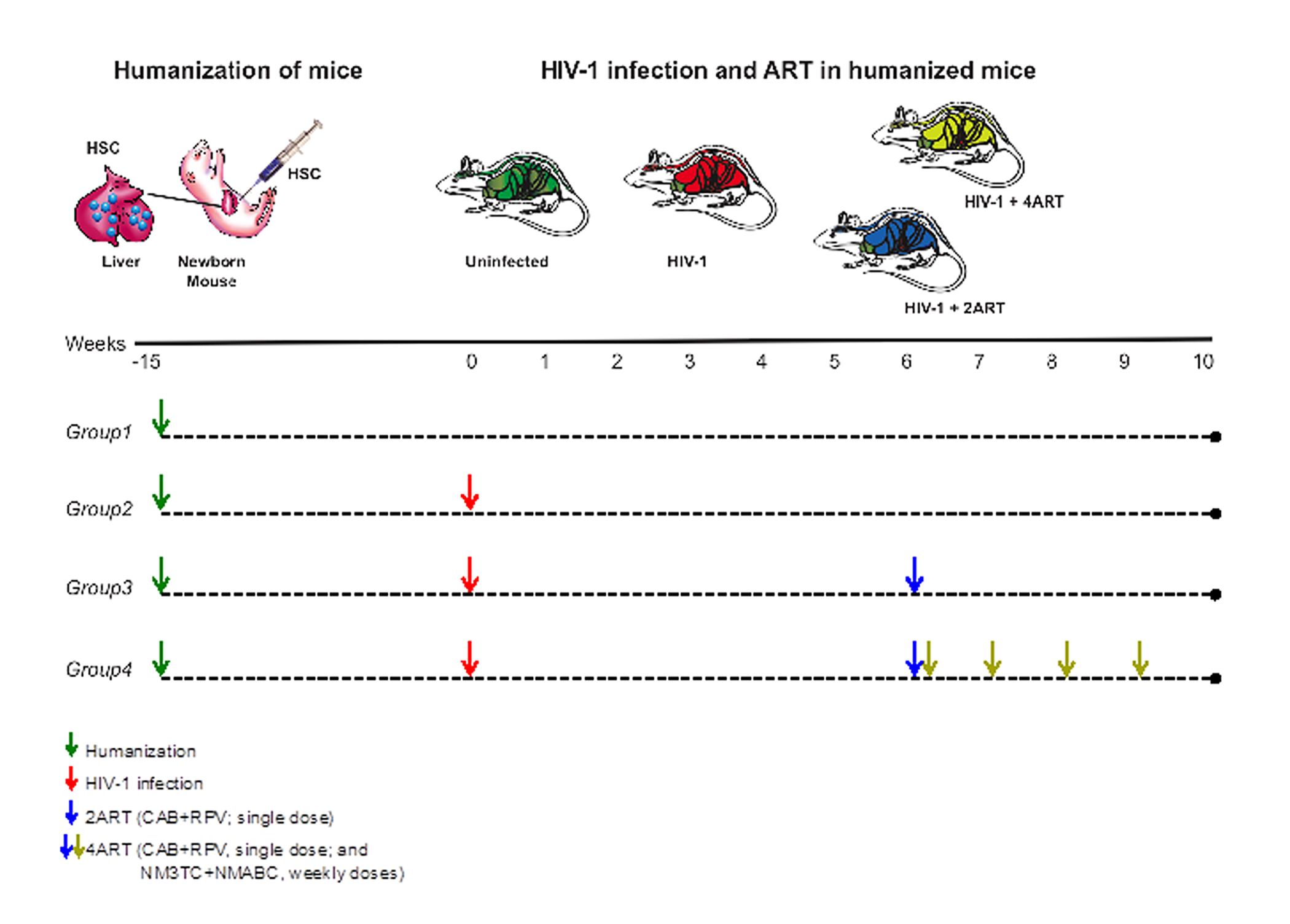

Supplement: Supplementary file 1 — Additional file 1: Figure S1 Antiretroviral treatment scheme used in HIV-1 infected humanized mice. [file 12977_2017_344_MOESM1_ESM.tif]
